# Nucleation, Development and Healing of Micro-Cracks in Shape Memory Polyurethane Subjected to Subsequent Tension Cycles

**DOI:** 10.3390/polym16131930

**Published:** 2024-07-06

**Authors:** Maria Staszczak, Leszek Urbański, Arkadiusz Gradys, Mariana Cristea, Elżbieta Alicja Pieczyska

**Affiliations:** 1 Institute of Fundamental Technological Research, Polish Academy of Sciences, 02-106 Warsaw, Poland; mstasz@ippt.pan.pl (M.S.); lurban@ippt.pan.pl (L.U.); agrad@ippt.pan.pl (A.G.); 2 “Petru Poni” Institute of Macromolecular Chemistry, 700487 Iași, Romania; mcristea@icmpp.ro

**Keywords:** polyurethane shape memory polymer, glass transition temperature, tensile loading cycles, structure analysis, micro-cracks, healing

## Abstract

Thermoresponsive shape memory polymers (SMPs) have garnered increasing interest for their exceptional ability to retain a temporary shape and recover the original configuration through temperature changes, making them promising in various applications. The SMP shape change and recovery that happen due to a combination of mechanical loading and appropriate temperatures are related to its particular microstructure. The deformation process leads to the formation and growth of micro-cracks in the SMP structure, whereas the subsequent heating over its glass transition temperature *T_g_* leads to the recovery of its original shape and properties. These processes also affect the SMP microstructure. In addition to the observed macroscopic shape recovery, the healing of micro-crazes and micro-cracks that have nucleated and developed during the loading occurs. Therefore, our study delves into the microscopic aspect, specifically addressing the healing of micro-cracks in the cyclic loading process. The proposed research concerns a thermoplastic polyurethane shape memory polymer (PU-SMP) MM4520 with a *T_g_* of 45 °C. The objective of the study is to investigate the effect of the number of tensile loading-unloading cycles and thermal shape recovery on the evolution of the PU-SMP microstructure. To this end, comprehensive research starting from structural characterization of the initial state and at various stages of the PU-SMP mechanical loading was conducted. Dynamic mechanical analysis (DMA), differential scanning calorimetry (DSC), wide-angle X-ray scattering (WAXS) and scanning electron microscopy (SEM) were used. Moreover, the shape memory behavior in the thermomechanical loading program was investigated. The obtained average shape fixity value was 99%, while the shape recovery was 92%, which confirmed good shape memory properties of the PU-SMP. Our findings reveal that even during a single loading-unloading tension cycle, crazes and cracks nucleate on the surface of the PU-SMP specimen, whereas the subsequent temperature-induced shape recovery process carried out at the temperature above *T_g_* enables the healing of micro-cracks. Interestingly, the surface of the specimen after three and five loading-unloading cycles did not exhibit crazes and cracks, although some traces of cracks were visible. The traces disappeared after exposing the material to heating at *T_g_* + 20 °C (65 °C) for 30 min. The crack closure phenomenon during deformation, even without heating over *T_g_*, occurred within three and five subsequent cycles of loading-unloading. Notably, in the case of eight loading-unloading cycles, cracks appeared on the surface of the PU-SMP and were healed only after thermal recovery at the particular temperature over *T_g_*. Upon reaching a critical number of cycles, the proper amount of energy required for crack propagation was attained, resulting in wide-open cracks on the material’s surface. It is worth noting that WAXS analysis did not indicate strong signs of typical highly ordered structures in the PU-SMP specimens in their initial state and after the loading history; however, some orientation after the cyclic deformation was observed.

## 1. Introduction

In recent years, shape memory polymers (SMPs) have become increasingly attractive as innovative smart materials, owing to their remarkable ability to memorize temporary shape and revert to their original configuration under specific conditions [1,2,3,4,5]. The shape memory effect (SME) in SMPs is triggered by exposure to various external stimuli, e.g., heat [6,7,8,9], light [10,11,12], moisture [13,14], electric [15] and magnetic fields [16,17]. The SME results from a synergistic interplay of the material’s morphology, structure and specific programming processes, which together enable the polymer to exhibit shape memory behavior [1,18].

Among various SMPs, thermoresponsive shape memory polymers have gained significant attention. Their responsiveness to changes in temperature makes them promising in various applications, from aircraft and aerospace to biomedicine [19,20,21,22,23,24,25]. At the molecular level, thermo-responsive SMPs consist of molecular switching segments and net points. Net points determine the permanent shape, while switching segments facilitate the shape recovery process. The shape memory behavior of SMPs is associated with a transition temperature, which often corresponds to the glass transition temperature (*T_g_*). Consequently, a temporary shape achieved through deformation at the temperature above *T_g_* can be fixed by cooling the SMP to the temperature below *T_g_* and then restored by reheating to the temperature above *T_g_* [1,6,26].

Polyurethane shape memory polymers (PU-SMP) represent a class of thermoresponsive SMPs renowned for their remarkable shape memory behavior and adaptable properties [27,28,29,30,31,32]. Thermoplastic shape memory polyurethanes are widely used due to their combination of good mechanical properties, easy processability, relatively low cost and high degree of shape recovery [6,33,34]. These polymers are characterized by their multiblock copolymer structure of soft segments and hard segments, resulting in a two-phase microstructure where the soft phase predominates [35,36]. To date, PU-SMPs have been used in various applications, including textile and apparel products, medical devices and soft actuators [37,38,39,40].

The mechanical loading and deformation process leads to the nucleation and development of micro-cracks in the material structure and, as a consequence, can result in material failure. Developing strategies to prevent or heal the micro-cracks enables us to anticipate the material’s performance over time and mitigate the risk of failure [41].

The shape change of an SMP that happens due to mechanical loading at an appropriate temperature is related to its particular microstructure. The subsequent heating of the SMP specimen causes the recovery of its original shape and also affects its microstructure. In addition to the observed macroscopic shape recovery, the healing of micro-crazes and micro-cracks can occur. A self-healing mechanism is highly advantageous in various engineering applications, as it can prolong the lifespan of structures and minimize maintenance requirements [42,43,44].

The first approach for achieving self-healing in materials involves introducing healing agents within the material itself. These agents can migrate to the location of a crack and repair it. Hayes et al. incorporated a thermoplastic into a thermosetting resin [45]. During the heating of a fractured resin system, the thermoplastic material diffused throughout the thermosetting matrix, effectively closing the cracks and facilitating the healing mechanism. Boiko investigated the mechanism of self-healing of glassy amorphous polymers by heating [46]. The author noticed the healing of cracks occurred due to molecular diffusion across the interface between.

The other approach implies that self-healing can be accomplished by creating structures with characteristics that enable them to close cracks or gaps autonomously, without using external healing agents [47]. A commonly used method to achieve healing is applying heat to the damaged area and increasing the temperature above their *T_g_*. This process enables the surfaces to merge and repair, facilitating the movement and re-entanglement of the polymer chains. For example, Chen et al. synthesized two highly cross-linked polymers and studied their thermal, mechanical and healing properties [48]. They demonstrated that cracks can be efficiently repaired through the thermal healing process. Kim et al. reported the development of a transparent thermoplastic polyurethane that could self-heal at 25 °C within 2 h, recovering over 75% of its mechanical properties through aromatic disulfide metathesis [49]. Deng et al. developed a series of polyurethane networks and found that the polyurethane containing 12% disulfide bonds with a relatively low degree of cross-linking exhibited the highest healing efficiency, achieving 94% recovery at 55 °C over 12 h [50]. Li et al., investigated the healing efficiency of a thermosetting polymer composite embedded with thermoplastic shape memory polymer fibers and thermoplastic particles and the possibility of healing wide-opened cracks by localized heating surrounding the cracked region [51]. To the best of the authors’ knowledge, most studies on healing phenomena have used heating over *T_g_*, while the effect of cyclic deformation on the healing process without subsequent heating has not been investigated, especially in the thermoplastic shape memory polyurethane.

The goal of our research is to investigate the impact of tension loading-unloading cycles on the microstructure evolution of the thermoplastic PU-SMP with a *T_g_* of 45 °C. Thermal recovery has been also considered. This study delves into the microscopic aspect, specifically addressing the healing of micro-cracks that occurs on the PU-SMP surface during subsequent loading-unloading cycles. To this end, comprehensive research starting from structural characterization was conducted by dynamic mechanical analysis (DMA), differential scanning calorimetry DSC, wide-angle X-ray scattering WAXS and scanning electron microscopy SEM at various stages of the PU-SMP cyclic loading. Tension cyclic tests with one, three, five and eight loading-unloading cycles have been conducted. The surface of the investigated specimens after deformation as well as after the thermal recovery was observed by SEM and analyzed. The optimum number of loading-unloading cycles in which micro-cracks formed and healed in the PU-SMP specimen without subsequent heating was found. The effect of loading temperature on the mechanical behavior of the PU-SMP during tension in the thermal chamber was also investigated. To explore this, the tension loading-unloading was performed at three various temperatures: at a temperature below *T_g_* (25 °C), above *T_g_* (65 °C) and at a temperature equal to *T_g_* (45 °C). Moreover, the shape memory properties, namely shape fixity and shape recovery, were investigated in the program of thermomechanical loading.

The conducted study provides empirical data on the optimal conditions for micro-crack healing and establishes the groundwork for further investigations into applications of SMPs in fields requiring durable and self-healing materials and structures.

## 2. Materials and Methods

### 2.1. Materials and Specimens

**Figure 1:**
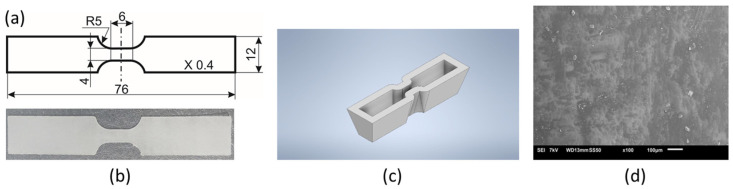
Shape and dimensions of a dogbone PU-SMP specimen: (**a**) drawing; (**b**) picture; (**c**) specially designed die cutter—3D drawing; (**d**) surface morphology at the initial state.

In the research, we have investigated the thermoplastic PU-SMP (designated by the producer as MM4520), purchased from SMP Technologies Inc., Tokyo, Japan. The glass transition temperature of the PU-SMP was equal to 45 °C. The dog bone specimens with dimensions of 75.6 × 12 × 0.4 mm, a gauge length of 6 mm and a gauge width of 4 mm (Figure 1a) were cut from a polymer sheet by a specially designed die cutter (Figure 1c). The shape and dimensions of the dog-bone specimens used for uniaxial tensile mechanical loading were determined based on numerous preliminary studies carried out by the authors on this material type. A photograph of the PU-SMP specimen is depicted in Figure 1b.

Before loading, all the specimens were heated at temperature *T_g_* + 20 °C = 65 °C for 30 min to obtain a uniform microstructure and erase the microstructural history that occurs during the material processing. Subsequently, the specimens were removed from the thermal chamber and allowed to cool to room temperature under ambient conditions.

The image of the surface of the PU-SMP with *T_g_* = 45 °C at the initial state before heating and deformation obtained by scanning electron microscopy is shown in Figure 1d. The presence of randomly dispersed irregularities on the surface of the material confirmed the micro-phase separation of PU-SMP, which occurs as a result of limited compatibility between the hard and soft segments [52,53].

### 2.2. Dynamic Mechanical Analysis (DMA)

**Figure 2:**
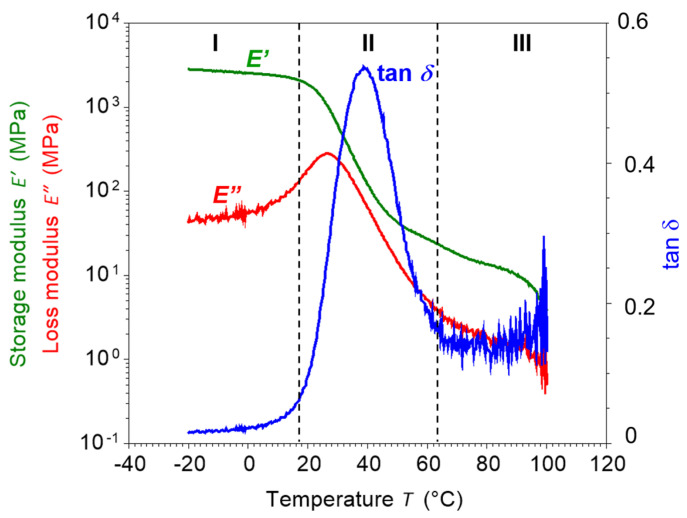
DMA result obtained for the PU-SMP: variation in storage modulus *E*′ (green color), loss modulus *E*″ (red color) and loss factor tan *δ* (blue color) with temperature. I, II and III denote the identified glassy, glass transition and rubbery regions, respectively.

Dynamic mechanical analysis (DMA) was conducted on an RSA-G2 analyzer (TA Instruments, New Castle, DE, USA) in tension mode to characterize the viscoelastic properties of PU-SMP. The bar shape specimens with a length of 15 mm, width of 10 mm and thickness of 0.4 mm were used. The experiments were run by increasing the temperature in ramp mode at a heating rate of 2 °C/min and a frequency of 1 Hz in the temperature range from −30 °C to 100 °C. The changes in the storage modulus (*E*′), loss modulus (*E*″) and loss factor (tan *δ*) were recorded as a function of temperature (Figure 2).

### 2.3. Thermogravimetric Analysis (TGA)

The thermogravimetric analysis of the samples was performed on a Discovery TGA 5500 (TA Instruments, New Castle, DE, USA) to determine the thermal stability of the PU-SMP. The tests were conducted in a nitrogen atmosphere at a flow rate of 25 mL/min. The samples with a weight of around 6 mg were placed in a platinum pan and were heated at a heating rate of 10 °C/min from ambient temperature to 700 °C.

### 2.4. Differential Scanning Calorimetry (DSC)

Differential scanning calorimetry (DSC) analysis was performed by a Discovery DSC 250 (TA Instruments, New Castle, DE, USA) under a nitrogen atmosphere in order to characterize the PU-SMP thermal response and to determine its transition temperatures. The enthalpy (cell) constant calibration was conducted using high-purity indium. A PU-SMP samples weighing approximately 6 mg were enclosed in standard aluminum pans. The procedure involved a heating-cooling-heating mode at a rate of 10 °C/min in the temperature range from 0 °C to 200 °C.

### 2.5. Investigation of Mechanical Properties

The mechanical investigation of the PU-SMP was carried out on the Instron 5969 testing machine (Instron, Norwood, MA, USA) coupled to the Instron thermal chamber 3119-606.

In order to characterize the PU-SMPs’ mechanical behavior and to choose an appropriate deformation range for cyclic loading, the program was started with the tension tests until rupture with a strain rate of 10^−2^ s^−1^ at room temperature. Afterwards, tension cyclic tests with one, three, five and eight subsequent loading-unloading cycles were conducted at the same strain rate of 10^−2^ s^−1^. Moreover, to investigate the effect of loading temperature on the PU-SMP mechanical behavior, the tension loading-unloading with the same strain rate was performed at three various temperatures: at a temperature below *T_g_* (25 °C), above *T_g_* (65 °C) and at a temperature equal to *T_g_* (45 °C). The repeatability of measurements was checked by performing at least three mechanical tests in each condition.

### 2.6. Investigation of Shape Memory Properties in the Thermomechanical Loading Program

The shape memory properties of the PU-SMP were investigated in the thermomechanical loading program, which was also performed in the thermal chamber of the Instron 5969 testing machine (Instron, Norwood, MA, USA). Typically, the thermal chamber temperature is measured with a single thermocouple near the air inlet, as designed by the Instron manufacturer. However, in order to increase the accuracy of our investigation, three additional thermocouples were introduced into the thermal chamber. One thermocouple was placed on the upper grip of the testing machine, another near the specimen gauge length and the third on the lower grip. After setting the desired temperature in the chamber, we monitored the temperatures from all three thermocouples. We began loading the PU-SMP specimen when the temperature difference between the thermocouples was less than 1 °C, ensuring a measurement accuracy within ±1 °C.

### 2.7. Scanning Electron Microscopy (SEM)

The surface of the investigated specimens before, after particular deformation programs and after subsequent heating was observed by a scanning electron microscope JEOL JSM-6010PLUS//LV InTouchScope™ (JEOL Ltd., Akishima, Japan). Before starting SEM imaging, all samples were sputter-coated with a gold layer for better conductivity using a SC7620 Polaron mini sputter coater (Quorum Technologies Ltd., Ashford, UK).

### 2.8. Wide-Angle X-ray Scattering (WAXS)

Wide-angle X-ray scattering investigations were conducted using Bruker D8 Discover Diffractometer (Mannheim, Germany). The measurements were conducted using Cu Kα radiation of wavelength λ = 1.5406 Å at a voltage of 40 kV and electrical current of 40 mA in transmission. Formation of the incident beam was performed using optics consisting of Göbel mirrors, 1 mm circular slit and 0.5 mm collimator. Images were recorded using a two-dimensional detector Vantec-500 located at 76.4 mm distance from the sample. Exact calibration of the detector-sample distance was performed using the silver behenate calibration standard. All measurements were conducted at ambient temperature, c.a. 23 °C. X-ray scattering images were recorded for 30 min.

## 3. Results and Discussion

### 3.1. Structural and Thermal Characterization Results

#### 3.1.1. Dynamic Mechanical Analysis Results

Figure 2 presents the DMA diagram obtained for the PU-SMP (MM4520), presenting the storage modulus *E*′, loss modulus *E*″ and loss factor tan *δ* vs. temperature. Three regions can be identified: I—glassy, II—glass transition and III—rubbery. The results obtained and outlined in Table 1 present the parameters influencing the shape memory characteristics of the PU-SMP. The parameters are instrumental in defining the material’s response to stimuli and its subsequent shape-recovery capabilities [54]. The obtained value of storage modulus in the glassy state *E*′*_g_* which was equal to 2550.0 MPa, was high. It ensures good fixity of the new polymer shape. The low value of storage modulus in the rubbery state *E*′*_r_*, which was equal to 25.6 MPa, facilitated a high deformation value and significant elastic recovery at higher temperatures. The high value of the obtained elasticity ratio of *E*′*_g_*/*E*′*_r_* (99.6) demonstrated a two-order-of-magnitude difference in elastic modulus values above and below *T_g_* and ensured a sharp transition from the glassy to the rubbery phase, rendering the material highly responsive to temperature changes. The glass transition temperature, as determined by the peak of the loss tangent tan *δ*, was equal to 38.8 °C.

#### 3.1.2. Thermogravimetric Analysis Results

**Figure 3:**
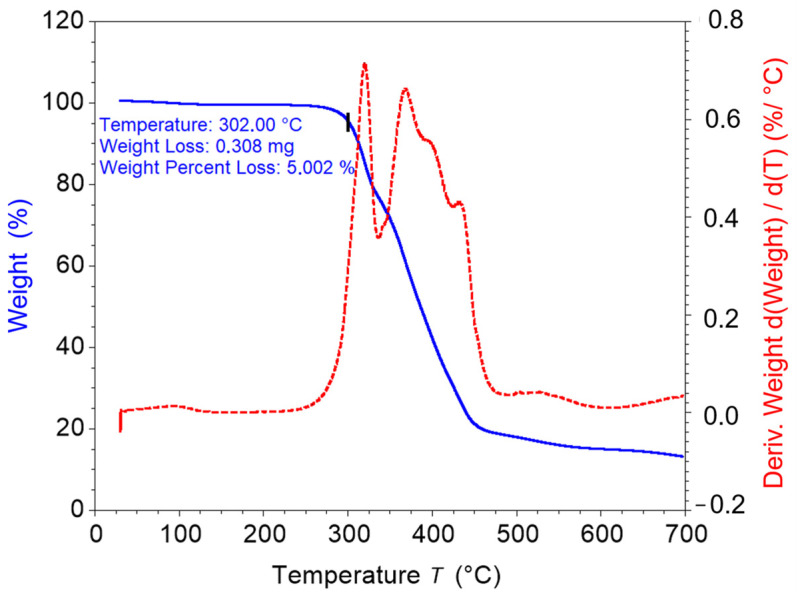
TGA curves, i.e., the weight percentage (blue line) and weight derivative (red line) vs. the temperature of the PU-SMP.

The obtained results of thermogravimetric analysis TGA−the weight percentage (blue line) and weight derivative (red line) vs. temperature curves−are shown in Figure 3.

According to Figure 3, the degradation of the PU-SMP began after 300 °C, and the temperature of degradation (*T_onset_*) was considered to be the temperature at which 5% of the initial weight was lost (302 °C). The thermal degradation took place in two steps, emphasized by the break point of the steep descent of the weight (blue line) and the two peaks of the derivative weight curve (red line). The first step was associated with the breaking of urethane bonds that were part of the hard domains. In the second step, the degradation of the soft phase, mainly comprised of marodiol, took place. However, the influence of the degradation products resulting from the first stage was evident as the second stage of the derivative weight curve consisted of overlapping peaks [55,56].

#### 3.1.3. Differential Scanning Calorimetry (DSC) Results

**Figure 4:**
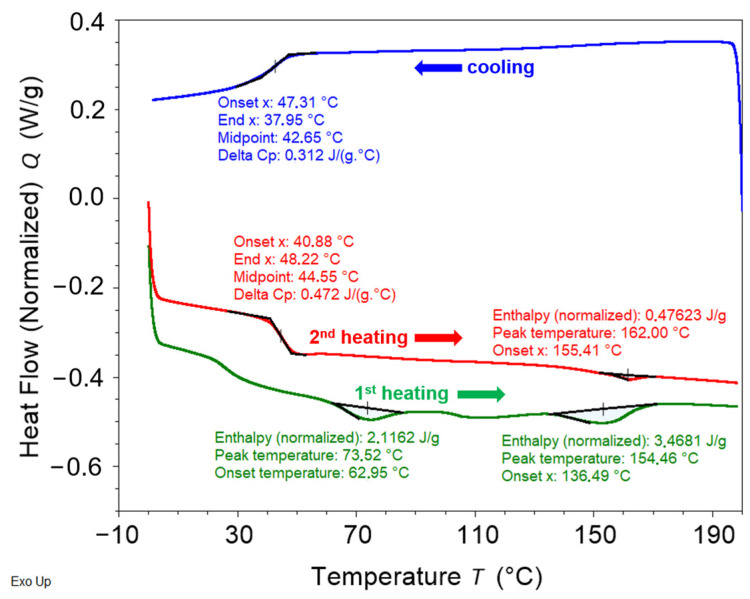
DSC curve of the PU-SMP.

The DSC curve, containing 1st heating, cooling and 2nd heating scans for the investigated PU-SMP at the initial state is shown in Figure 4. The 1st heating scan demonstrates two endothermic peaks located at c.a. 74 °C (2.12 J/g) and 154 °C (3.47 J/g), which can be associated with two different types of crystalline domains. The cooling scan shows *T_g_* at c.a. 42.7 °C. The 2nd heating scan shows *T_g_* at 44.6 °C followed by a small endothermic melting peak at c.a. 162 °C (0.48 J/g).

It should be taken into account that usually the glass transition temperature obtained during the 2nd heating is more reliable. The microstructure of the specimen may have been changed during the processing and the 2nd heating scan reflects the state of the sample structure after the history has been erased during the 1st heating. Therefore, the glass transition temperature was determined from the second heating and it was equal to 44.5 °C. The obtained temperature is similar to those, given by the PU–SMP producer.

### 3.2. Results and Analysis of Mechanical Characteristics of the PU–SMP with T_g_ = 45 °C

#### 3.2.1. Mechanical Behavior of PU–SMP during Tension until Rupture

**Figure 5:**
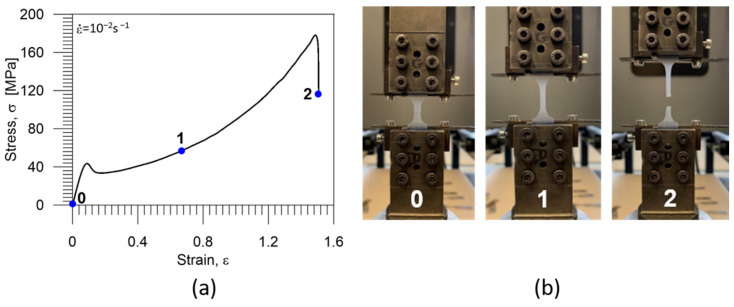
(**a**) Stress—strain curve of the PU-SMP obtained during tension till rupture; (**b**) photographs showing subsequent stages of specimen deformation: 0—before loading, 1—during the advanced stage of loading, 2—just after rupture.

In order to learn more about the mechanical behavior of the studied PU–SMP, a tension loading with the strain rate of 10^−2^ s^−1^ until rupture at room temperature was conducted. The obtained average stress-strain curve is presented in Figure 5a. The elongation at the break of the polymer was about 150%. Based on these results, the particular strain value beyond the yield limit for cyclic tests was chosen as 60%. On the right side of the diagram, three photographs taken at various strain values marked in Figure 5a and depicting the subsequent stages of the PU-SMP tension are presented: 0—before loading, 1—during the advanced stage of loading, 2—just after the specimen rupture (Figure 5b).

It can be seen in Figure 5b (1) that an optical whitening effect occurred during the specimen deformation. In terms of the PU–SMP structure evolution, it may be a result of the reorientation of the polyurethane molecular chains that straighten when stretched. Moreover, voids and microcracks also appeared in the polymer under loading [57]. When the sizes of the microcracks were larger than the wavelength of light, light scattered on the microcracks and the refractive index changes, making the object appear white.

#### 3.2.2. Mechanical Characteristics of the PU-SMP with *T_g_* = 45 °C Subjected to Tension at Various Temperatures in the Strain Range of 60%

**Figure 6:**
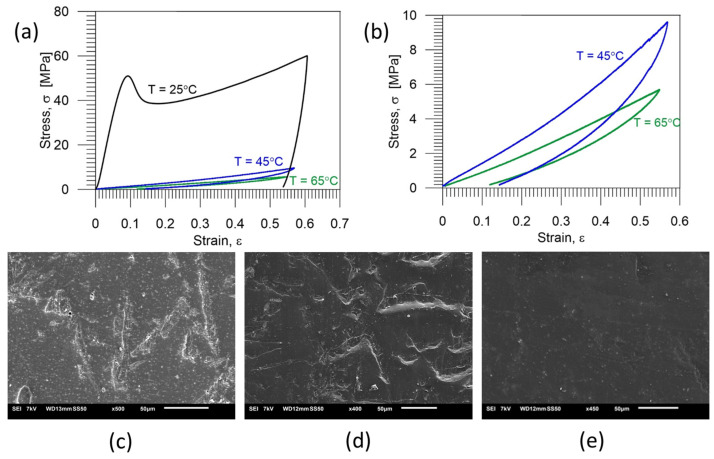
(**a**) Stress-strain curves of the PU-SMP obtained during one-cycle loading-unloading at a strain rate of 10^−2^ s^−1^ in the strain range of 60% at various temperatures: below *T_g_* (25 °C), around *T_g_* (45 °C) and above *T_g_* (65 °C); (**b**) stress-strain curves obtained at *T_g_* and *T_g_* + 20 °C with a larger scale. Below, the PU-SMP microstructure obtained after one loading-unloading tension cycle is shown at the temperature of: (**c**) below *T_g_* (25 °C)*, (**d**) around *T_g_* (45 °C)* and (**e**) above *T_g_* (65 °C)*. * The sample surface for SEM investigation was prepared, and the SEM image was recorded and analysed by the IPPT PhD students—Mana Nabavian Kalat and Yasamin Ziai.

The PU-SMP mechanical behavior at various temperatures was also investigated. To explore this, tension loading-unloading tests with a strain rate of 10^−2^ s^−1^ up to approximately 60% strain were performed at three various temperatures: below *T_g_* (25 °C), equal to *T_g_* (45 °C) and at the temperature above *T_g_* (65 °C). A comparison of the average stress-strain curves at various temperatures is depicted in Figure 6a. For better visualization, stress-strain curves obtained at *T_g_* and at *T_g_* + 20 °C are also presented on a larger scale in Figure 6b. The estimated PU-SMP mechanical parameters are provided in Table 2.

At temperatures below the *T_g_*, the polymer was in a glassy state and was characterized by a high elastic modulus (718.81 MPa), which was the result of frozen micro-Brownian motion, e.g., [5]. The stress-strain behavior of PU–SMP resembled those demonstrated by elastoplastic materials. Initially, there was a reversible, linear deformation region indicating elastic behavior, featured by small strain and insignificant shape changes. Subsequently, the plastic stage ensued, involving irreversible polymer deformation mechanisms of a dissipative nature. The linear segment reached its peak stress, signifying the yield point, followed by stress reduction due to strain localization phenomena. As strain increased, stress levels escalated further. Finally, the damage occurred, which was associated with polymer chain breakage resulting in the specimen rupture [26].

By comparing the stress-strain characteristics obtained at various temperatures, it can be noticed that stress values and Young’s moduli at *T_g_* and above *T_g_* were much lower (*E* = 13.35 MPa at *T_g_*; *E* = 8.94 MPa at *T_g_* + 20 °C) than those obtained at a temperature below *T_g_*. Moreover, at *T_g_* and above *T_g_*, distinct yield points were not evident. Near *T_g_*, the PU-SMP transitioned into a rubbery state in which the soft segment movements were activated [6]. This state was characterized by higher entropy and the related flexibility of the polymer chains, facilitating easy deformation and resulting in lower stress values.

The images of the microstructure after the tension loading-unloading cycle at 25 °C, 45 °C and 65 °C are presented in Figure 6c–e, respectively. The cracks were observed only in the case of loading at a temperature below *T_g_*. When the PU–SMP specimen was loaded at the *T_g_* and above the *T_g_*, the PU–SMP surface was smooth, without any cracks (Figure 6d,e).

### 3.3. Investigation of PU-SMP Shape Memory Properties

The shape memory properties of PU-SMP were investigated in the thermomechanical program. In the initial stage, the specimen was heated to 65 °C (*T_g_* + 20 °C) at 12 °C/min. Then, tension loading (stage I) was applied with a strain rate of 10^−3^ s^−1^ at 65 °C until a maximum programmed strain of 20% (*ε_m_*) was reached. Next, the specimen was cooled to room temperature (25 °C) while maintaining the maximum strain (*ε_m_*) (stage II) to fix its temporary shape. Subsequently, the specimen was unloaded (stage III) to zero-force at 25 °C with a strain rate of 10^−3^ s^−1^, demonstrating the fixity of the new shape. Finally, the specimen was reheated from room temperature to *T_h_* = 65 °C (*T_g_* + 20 °C) at a heating rate of 12 °C/min under no-load conditions, leading to the recovery of the PU-SMP’s original shape (stage IV). The experiments were conducted on four specimens to confirm the repeatability of the results and to thoroughly evaluate the shape memory properties.

**Figure 7:**
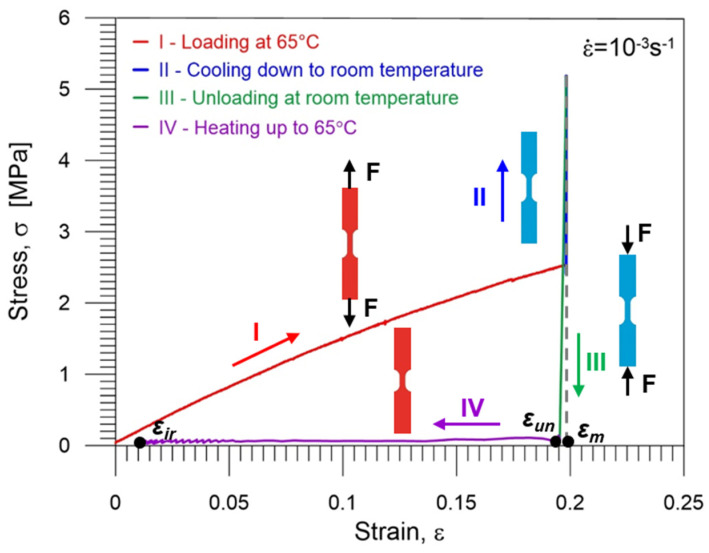
Stress vs. strain curve obtained during the PU-SMP thermomechanical loading program; particular colors of curves mark subsequent stages of the program: I (red)—loading at 65 °C (*T_g_* + 20 °C), II (blue)—cooling down to room temperature (*T_g_* − 20 °C) while the stress increases, III (green)—unloading at room temperature to zero stress, IV (violet)—second heating up to 65 °C (*T_g_* + 20 °C) and shape recovery. Schematic demonstration of the specimen at the subsequent stages of thermomechanical loading (I–IV) shows its shape and gauge length changes.

The stress vs. strain curve obtained during the thermomechanical loading cycle is depicted in Figure 7. Different colors in the diagram represent distinct stages of the process. Key strain values (*ε_m_*, *ε_un_* and *ε_ir_*) essential for the calculation of the shape memory parameters are marked by black dots. It is evident from Figure 7 that the strain value after unloading *ε_un_* closely approximates the maximum strain value *ε_m_*, highlighting the PU-SMP’s high shape fixity property. Upon reheating above *T_g_*, the PU-SMP specimen reverted to its initial shape; however, some small irrecoverable strain *ε_ir_* was observed.

The first heating above *T_g_* increased the flexibility in the soft segments of the polymer chains, enhancing bond rotations and system entropy. This created more macromolecular conformations and random coils. Application of even slight loads caused polymer chains to disentangle, reducing their mobility and resulting in a significant entropy decrease. Cooling below *T_g_* restricted chain movement, fixing the temporary shape upon constraint removal. Next, heating above *T_g_* restored random coil configurations, returning the polymer to a high entropy state and allowing recovery of the original shape of the PU-SMP [1, 8, 31].

The PU-SMP shape fixity *R_f_* and shape recovery *R_r_* parameters essential for applications were calculated using the obtained experimental data, according to Equations (1) and (2), proposed by H. Tobushi and S. Hayashi [58]:

(1) $${\;R}_{f}=\frac{\varepsilon_{un}}{\varepsilon_{m}}\cdot 100\%\;,$$

(2) $$R_{r}=\frac{\varepsilon_{m}-\varepsilon_{ir}}{\varepsilon_{m}}\cdot 100\%\;,$$

where *ε_m_*—the maximum strain, *ε_un_*—the strain obtained after unloading and *ε_ir_*—the irrecoverable strain obtained after heating to *T_g_* + 20 °C under no-load conditions.

The PU-SMP shape fixity and recovery parameters obtained for four specimens are summarized in Table 3, revealing non-significant differences among the results.

The thermoplastic PU-SMP with *T_g_* = 45 °C exhibited an average shape fixity of approximately 99%, indicating its high ability to maintain temporary shapes. The average shape recovery of approximately 92% suggested that while the material’s recovery was slightly lower than its fixity, it remained effective for ensuring reliable performance in shape memory applications.

### 3.4. Cyclic Loading—Mechanical and SEM Results

**Figure 8:**
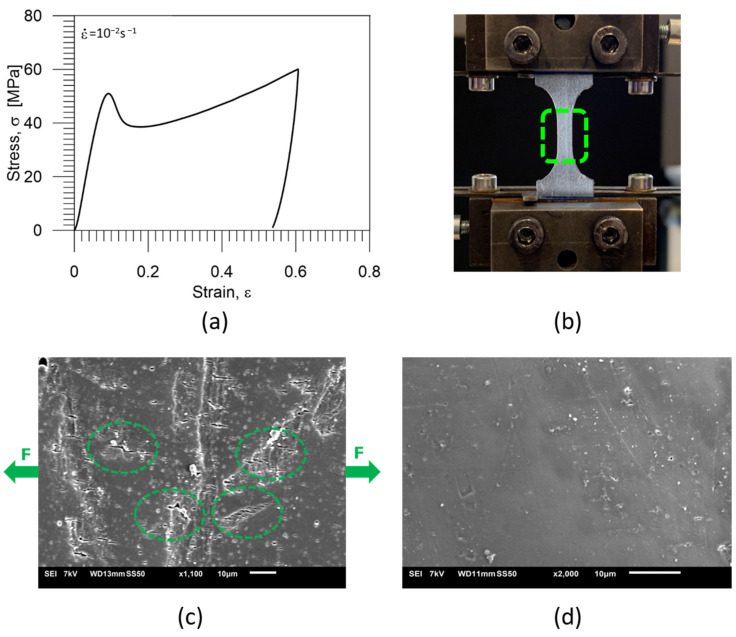
(**a**) Stress-strain curve of the PU-SMP specimen after one loading-unloading cycle, (**b**) a picture of the specimen in the grips of the testing machine showing the specific area of the specimen subjected to SEM investigation, (**c**) the microstructure after one-cycle loading-unloading* and (**d**) the microstructure of the PU-SMP specimen after one-cycle loading-unloading and thermal recovery at *T_g_* + 20 °C = 65 °C*. * The sample surface for SEM investigation was prepared, and the SEM image was recorded and analysed by the IPPT PhD students—Mana Nabavian Kalat and Yasamin Ziai.

PU-SMP specimens were subjected to cyclic tension loading-unloading at room temperature. The maximum loading strain was kept constant for each cycle at 60%, and afterwards, the strain was driven back until the stress-free state was reached. The formation and healing of micro-cracks were investigated through microscopic observation by SEM after the deformation and after the healing process that occurred during heating above *T_g_*. The specific area of the specimen subjected to SEM is shown in Figure 8b.

The obtained stress-strain curve after one loading-unloading cycle is presented in Figure 8a. The microstructure of the PU-SMP after deformation and after thermal healing are depicted in Figure 8c,d, respectively.

As seen in Figure 8c, by inducing one-cycle tensile loading-unloading, the deformation led to the nucleation and evolution of crazes and micro-cracks on the surface of the PU-SMP specimen. The micro−cracks appeared in line with the loading direction, denoted by green arrows. However, by further heating the specimen at *T_g_* + 20 °C = 65 °C, the thermal recovery occurred, and the cracks were healed (Figure 8d).

**Figure 9:**
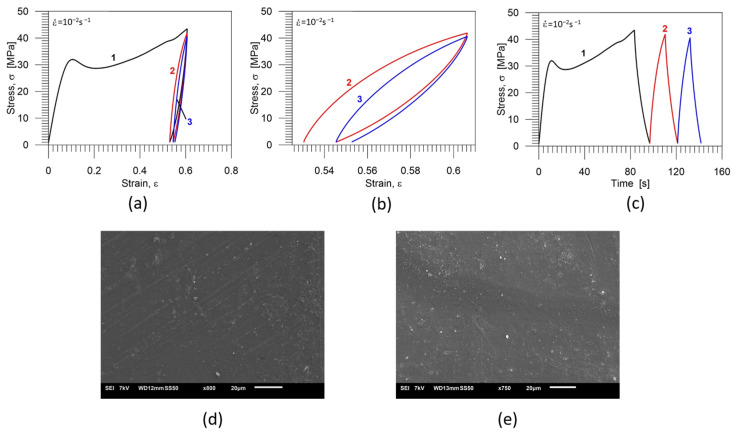
Mechanical characteristics of the PU-SMP specimen obtained for three-cycle loading-unloading program: (**a**) stress-strain curves; (**b**) stress-strain curves showing cycles 2 and 3 on a larger scale; (**c**) stress-time curves; particular colors denote subsequent cycles: black—1st cycle, red—2nd cycle; blue—3rd cycle; (**d**) the microstructure after three-cycle loading-unloading* and (**e**) the microstructure after the deformation and the thermal recovery*. * The sample surface for SEM investigation was prepared, and the SEM image was recorded and analysed by the IPPT PhD students—Mana Nabavian Kalat and Yasamin Ziai.

The stress-strain curves obtained for three-cycle loading-unloading are shown in Figure 9a; the stress-strain curve demonstrating only cycles 2 and 3 on a larger scale is presented in Figure 9b, while the stress vs. time curves are shown in Figure 9c. Particular colors denote subsequent cycles: black−first cycle, red−second cycle; blue−third cycle. The images of the PU-SMP microstructure obtained after three loading-unloading cycles and after deformation and thermal recovery are presented in Figure 9d,e, respectively.

In Figure 9d, except for some traces of cracks, no cracks or crazes were visible. After the thermal shape recovery of this specimen at 65 °C, even the crack’s traces disappeared, and the surface of the specimen looked smoother (Figure 9e).

**Figure 10:**
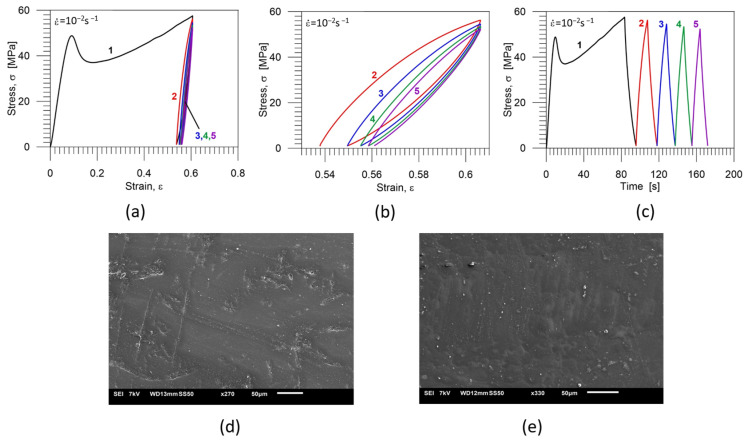
Mechanical characteristics of the PU-SMP specimen obtained for five-cycle loading-unloading program: (**a**) stress-strain curves; (**b**) stress-strain curves showing cycles 2, 3, 4 and 5 on a larger scale; (**c**) stress vs. time curves; particular colors denote subsequent cycles: black—1st cycle, red—2nd cycle; blue—3rd cycle, green—4th cycle, purple—5th cycle; (**d**) the microstructure after five loading-unloading cycles*; (**e**) the microstructure after the deformation and the thermal recovery*. * The sample surface for SEM investigation was prepared, and the SEM image was recorded and analysed by the IPPT PhD students—Mana Nabavian Kalat and Yasamin Ziai.

The stress-strain curves obtained for five-cycle loading-unloading program are shown in Figure 10a; the stress-strain curves demonstrating cycles 2, 3, 4 and 5 are presented on a larger scale in Figure 10b, and the stress vs. time curves are shown in Figure 10c. Particular colors denote subsequent cycles: black—first cycle, red—second cycle; blue−third cycle, green—fourth cycle, purple—fifth cycle. The images of the PU-SMP microstructure after the five—cycle loading-unloading program and after the thermal recovery are presented in Figure 10d,e, respectively.

In the case of five-cyclic loading-unloading program, except for some traces of cracks, no cracks or crazes were visible (Figure 10d). After the thermal shape recovery of the specimen at the temperature of *T_g_* + 20 °C = 65 °C, even the traces disappeared, and the surface of the specimen looked smoother (Figure 10e).

**Figure 11:**
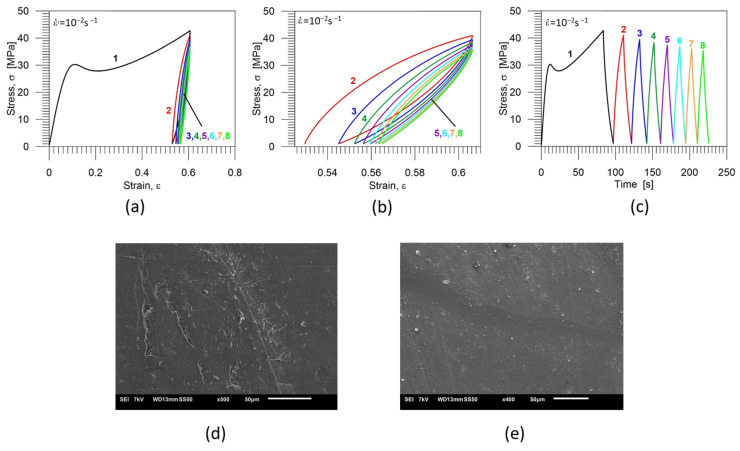
Mechanical characteristics obtained for the PU-SMP specimen subjected to eight loading-unloading cycles: (**a**) stress-strain curves; (**b**) stress-strain curves showing cycles 2, 3, 4, 5, 6, 7 and 8 on a larger scale; (**c**) stress-time curves; particular colors denote subsequent cycles: black—1st cycle, red—2nd cycle; blue—3rd cycle, green—4th cycle, purple—5th cycle, light blue—6th cycle, orange—7th cycle, light green—8th cycle; (**d**) the microstructure after eight-cycle loading-unloading* and (**e**) the microstructure after the deformation and the thermal recovery that occurred during subsequent heating at *T_g_* + 20 °C*. * The sample surface for SEM investigation was prepared, and the SEM image was recorded and analysed by the IPPT PhD students—Mana Nabavian Kalat and Yasamin Ziai.

The stress−strain curves obtained for the specimen subjected to eight loading-unloading cycles are shown in Figure 11a; the stress−strain curve demonstrating cycles 2–8 on a larger scale is presented in Figure 11b, while the stress vs. time curves is presented in Figure 11c. Particular colors denote subsequent cycles: black-first cycle, red-second cycle; blue-third cycle, green-fourth cycle, purple-fifth cycle, light blue-sixth cycle, orange-seventh cycle, light green-eighth cycle. The images of the PU-SMP microstructure after eight-cycle loading-unloading program, as well as after thermal recovery, are presented in Figure 11d,e, respectively.

The presence of micro−cracks after eight loading-unloading cycles on the surface of the PU-SMP was clearly observable (Figure 11d). However, during subjecting the specimen to heating at a temperature significantly above *T_g_* (*T_g_* + 20 °C = 65 °C), the thermal shape recovery of the specimen occurred, and the cracks were healed (Figure 11e).

Shape memory polymers can recover permanent plastic deformation by being exposed to external stimuli. However, it is not known in detail if they are also microscopically recovered. Polymers subjected to loading exhibit distinct behaviors in the structure evolution that can be in some approaches compared to metals [41].

The micromechanism of deformation that can occur in polymers is a craze yielding. Craze yielding is characterized by a highly localized plastic deformation process that leads to brittle failure. This phenomenon is commonly observed in amorphous and thermoplastic polymers [59].

**Figure 12:**
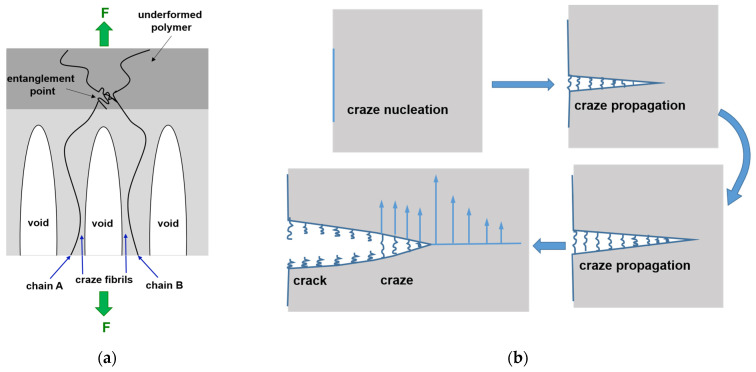
(**a**) Schematic representation of the stretching mechanism during the craze growth; (**b**) subsequent stages of formation of a crack from craze growth.

A schematic representation of the stretching mechanism during the craze growth is shown in Figure 12a. Crazes grow along a plane perpendicular to the tensile stress. A craze consists of fibrils that are separated by voids. Fibrils are aligned with the tensile stress. Chains A and B are entangled in the undeformed polymer layer. This entanglement point should be broken for the chains to continue to move into the fibrils. The increase in local stress triggers the failure of stretched fibrils spanning the craze, resulting in void coalescence and the propagation of cracks [59].

Generally, the nucleation of a craze can be facilitated by the existence of an initial craze, some defects or irregularities in the structure under the stress conditions. Breaking of the fibrils between the voids turns crazes into wide−open cracks (Figure 12b).

The observed phenomenon of closing wide-open cracks has been explained by Li et al. [51]. In the case of the studied PU-SMP, the crazes and cracks were nucleated even during the first loading-unloading cycle. According to the SEM image of the sample surface after one cycle of loading-unloading, wide-open cracks were formed during the deformation process. The subsequent heating at a temperature above *T_g_* led to the constrained shrinkage of the PU-SMP, resulting in the closure of the crack. Further heating leads to the melting of the soft segments. By increasing the number of cycles to three, the crack closure during deformation took place. Two free surfaces of the voids and crazes touched each other during the repeating of the loading-unloading cycle due to large deformation in the specimen plastic zone. The surfaces of the created formerly crazes and voids were glued back together. Consequently, the crack closure was an effect of plasticity.

It is also possible that upon reaching a critical number of the loading cycles, a sufficient amount of energy for crack propagation was accumulated, leading to the formation of wide-opened cracks on the material’s surface, as was observed in the case of eight cycles.

#### Cyclic Loading—Mechanical and Wide-Angle X-ray Scattering Results

**Figure 13:**
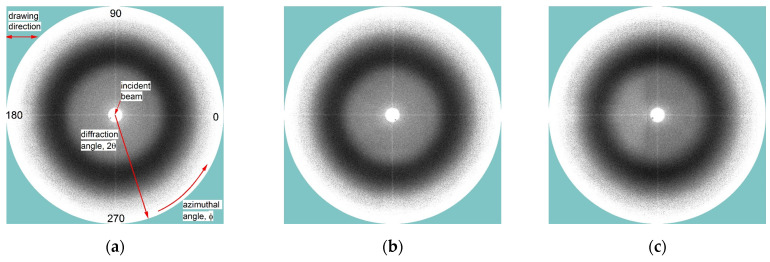
WAXS images of PU-SMP samples: (**a**) in the initial state, (**b**) after three loading-unloading cycles and (**c**) after eight loading-unloading cycles. White color denotes low intensity; black color—high intensity. In Figure 13a, there are also labeled directions of drawing, diffraction angle *2θ*, azimuthal angle *ϕ* and the position of the incident beam.

In order to examine whether the investigated PU-SMP exhibited signs of typical highly ordered structures, an investigation using the WAXS technique was performed on a sample as received (original) and samples after deformation by three and eight loading-unloading cycles. The obtained X-ray scattering images are presented in Figure 13.

The WAXS images presented in Figure 13 demonstrate mainly an intense broad ring resulting from the scattering of the incident beam by the amorphous phase, i.e., the amorphous halo [60]. The obtained WAXS images do not show any strong signs of typical highly ordered crystalline structures, which are usually observed as a set of narrow rings or arcs resulting from diffraction of the beam on specific crystal planes.

The observable differences in images obtained for the samples after three-cycle loading-unloading program (Figure 13b) and after eight-cycle loading-unloading (Figure 13c) compared to the sample in the initial state (Figure 13a) are related to the relative changes in the intensity of the scattering along the azimuthal angle. It is most evidently noticed for the sample after eight loading-unloading cycles at low diffraction angles 2*θ* < 15° while changing the azimuthal angle *ϕ* from 0° to 90° (Figure 13c).

**Figure 14:**
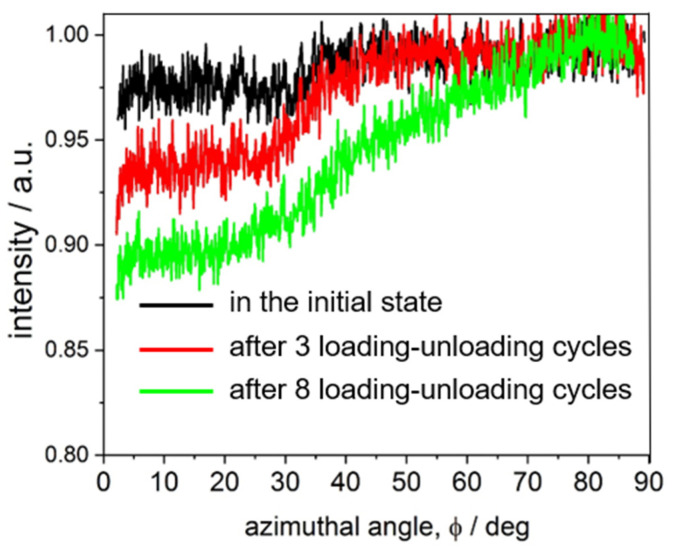
Results of the WAXS investigation: azimuthal distribution of the intensity, normalized.

Integration of the images over the diffraction angle *2θ* in the range from 5° to 28° plotted versus the azimuthal angle *ϕ* evidences the changes in the intensity, which is demonstrated in Figure 14.

As can be seen in Figure 14, the changes in the intensity for the deformed samples after three- and eight-cycle loading-unloading were weak; however, they indicate the presence of a small orientation in the samples after such loading program.

The orientation of the investigated samples’ structure was quantitatively characterized by Hermans orientation factor *f_a_*, commonly used in the case of crystalline samples [61]:

(3) $$f_{a}=\frac{1}{2}\left(3\langle \cos^{2}\varphi \rangle -1\right).$$

Meanwhile, $\langle \cos^{2}\varphi \rangle$ represents the mean-square cosine of the angle between some reference direction and a given crystal axis [Alexander, 1969]:

(4) $$\langle \cos^{2}\varphi \rangle =\frac{\int_{0}^{\pi /2}I\left(\varphi \right)\sin\varphi \cos^{2}\varphi d\varphi }{\int_{0}^{\pi /2}I\left(\varphi \right)\sin\varphi d\varphi }.$$

In the case of PU-SMP samples, as there were no crystals, $\langle \cos^{2}\varphi \rangle$ was treated as mean-square cosine of the angle between the drawing direction *ϕ* = 0° and the strongest scattering direction, which was observed in the perpendicular direction to the drawing direction, i.e., *ϕ* = 90°.

Basically, the Hermans factor takes values from 1, which is an ideal case when the orientation is parallel to the reference direction, up to −0.5, when the orientation is perpendicular to the reference (drawing) direction. If *f_a_* = 0, there is no preferred orientation or, in other words, there is random orientation. In the case of investigated samples, *f_a_* takes values around 0; in detail, for the sample in the initial state *f_a_* = 3 × 10^−3^, while for the deformed samples, *f_a_* was as follows: by three loading-unloading cycles, *f_a_* = −5 × 10^−4^, and by eight loading-unloading cycles, *f_a_* = 7 × 10^−3^, indicating that using the Hermans factor was rather not possible to detect the presence of any orientation in these samples.

**Figure 15:**
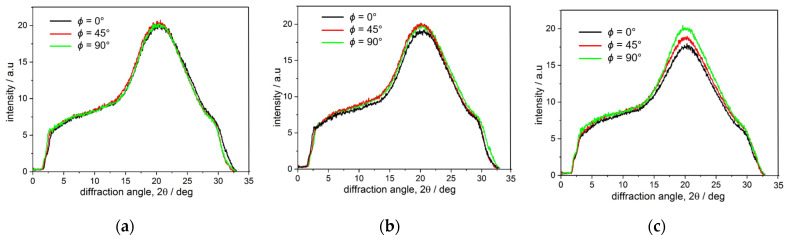
Intensity profiles over the diffraction angle *2θ* along the loading direction *ϕ* = 0°, perpendicular to the loading direction *ϕ* = 90° and in the diagonal direction *ϕ* = 45° determined from WAXS images (Figure 13) for PU-SMP samples: (**a**) in the initial state, (**b**) after three loading-unloading cycles, (**c**) after eight loading-unloading cycles. Intensity is integrated over the *ϕ −* 15°/*ϕ* + 15° range.

The diffraction profiles determined at the azimuthal angles *ϕ* equal to 0°, 45° and 90° are depicted in Figure 15.

It is demonstrated in Figure 15 that in the case of the sample at the initial state, there was no difference in the intensity profiles at the selected *ϕ* angles, indicating a complete lack of any orientation. However, Figure 15b,c evidence changes in the intensity of the amorphous halo with the maximum at *2θ* = 20° at selected *ϕ* angles. The strongest intensity changes were seen for the sample after eight loading-unloading cycles (Figure 15c), whereas in the case of the sample after three loading-unloading cycles (Figure 15b), the changes in intensity were the least evident.

## 4. Conclusions

Comprehensive research on the polyurethane shape memory polymer was conducted, starting from structural characterization using various techniques, e.g., dynamic mechanical analysis, differential scanning calorimetry, wide-angle X-ray scattering or scanning electron microscopy at various stages of the mechanical and thermal loadings.

The shape memory properties of the thermoplastic PU-SMP with *T_g_* = 45 °C were investigated in the program of thermomechanical loading, revealing an average shape fixity of approximately 99%, highlighting its strong capability to retain temporary shapes. The average shape recovery of approximately 92% indicates a slightly lower shape recovery rate compared to shape fixity, yet it ensures dependable performance in shape memory applications. These findings underscore the material’s suitability for applications requiring precise shape retention and reliable recovery mechanisms.

Tension cyclic tests of the PU-SMP were performed with one, three, five and eight loading-unloading cycles, followed by microstructural observation of the specimen surface after deformation and after thermal recovery by heating significantly above the glass transition temperature. The influence of the number of cycles on the mechanical behavior and the evolution of micro-cracks on the surface of the PU-SMP specimen was investigated.

Our findings reveal that even after a single loading-unloading cycle, crazes and cracks nucleate on the PU-SMP specimen’s surface. Furthermore, it has been noticed that the subsequent temperature-induced shape recovery process conducted at *T_g_* + 20 °C, enables macroscopic shape recovery as well as the healing of micro-cracks.

Interestingly, the surface of the specimen after three and five loading-unloading cycles did not contain crazes and cracks, although some traces of cracks were visible. These traces disappeared after exposing the material to heating significantly above *T_g_* (65 °C), resulting in a smooth PU-SMP surface.

This means that the cracks closure phenomenon happened even without the subsequent heating but during three or five loading-unloading cycles. Notably, cracks that appeared on the surface of the PU-SMP sample after eight loading-unloading cycles could be healed only during the thermal recovery process. Probably, upon reaching a critical number of cycles, the proper amount of energy required for crack propagation was attained, resulting in wide-opened cracks on the SMP sample’s surface.

WAXS analysis did not indicate strong signs of typical highly ordered structures in the PU-SMP specimens in the initial state and after the loading history. However, some orientation after higher cyclic deformation was observed.

The conducted research program embraces numerous experimental results aimed at investigating the optimal conditions for micro-crack healing phenomena towards prospective PU-SMP applications.

Future research that will explore other deformation modes and the influence of a higher number of loading-unloading cycles in various conditions on the PU-SMP mechanical behavior and structural changes is considered.

## Figures and Tables

**Table 1:** DMA results obtained for PU-SMP MM4520.

*E*′*_g_*, MPa	*E*′*_r_*, MPa	*E*′*_g_*/*E*′*_r_*	*T_g_* as tan *δ* Peak, °C
2550.0	25.6	99.6	38.8

**Table 2:** Mechanical parameters of PU–SMP *T_g_* 45 °C obtained during tension with a strain rate of 10^−2^ s^−1^ in the strain range of about 60% at 25 °C, 45 °C and 65 °C.

Loading Temperature, °C	Young’s Modulus, MPa	Yield Strength, MPa	Stress at Maximum Strain, MPa
25	718.81	50.73	58.97
45	13.35	-	9.59
65	8.94	-	5.67

**Table 3:** The shape fixity and shape recovery parameters obtained for the PU-SMP with *T_g_* = 45 °C in the thermomechanical loading program.

Specimen	Shape Fixity *R_f_,* %	Shape Recovery *R_r_,* %
1	98.75	93.34
2	98.62	91.64
3	98.63	90.96
4	98.64	92.32
Average	98.63 ± 0.01	92.06 ± 1.01

## Data Availability

Data are contained within the article.
