# Correction: Staszczak et al. Nucleation, Development and Healing of Micro-Cracks in Shape Memory Polyurethane Subjected to Subsequent Tension Cycles. *Polymers* 2024, *16*, 1930

**DOI:** 10.3390/polym17050560

**Published:** 2025-02-20

**Authors:** Maria Staszczak, Leszek Urbański, Arkadiusz Gradys, Mariana Cristea, Elżbieta Alicja Pieczyska

**Affiliations:** 1 Institute of Fundamental Technological Research, Polish Academy of Sciences, 02-106 Warsaw, Poland; mstasz@ippt.pan.pl (M.S.); lurban@ippt.pan.pl (L.U.);; 2 “Petru Poni” Institute of Macromolecular Chemistry, 700487 Iași, Romania; mcristea@icmpp.ro

In the original publication [1], the proposed text in the Acknowledgments Section did not properly reflect Mana Nabavian Kalat and Yasamin Ziai’s role in acquiring and analyzing the SEM images of the PU-SMP specimens.

The following statement was added to the figure captions with an asterisk at the end of each figure (Figures 6c–e, 8c,d, 9d,e, 10d,e and 11d,e):

“The sample surface for SEM investigation was prepared, and the SEM image was recorded and analysed by the IPPT PhD students—Mana Nabavian Kalat and Yasamin Ziai”.

The Acknowledgments Section was modified to the following: 

“The authors express their thanks to Mana Nabavian Kalat and Yasamin Ziai for acquiring and analysing the SEM results presented in Figures 6c–e, 8c,d, 9d,e, 10d,e and 11d,e. We would also like to extend our thanks to Mana Nabavian Kalat for her assistance during the mechanical testing. The authors extend their sincere appreciation to Hisaaki Tobushi and Kohei Takeda for their valuable guidance on the PU-SMP mechanical and thermomechanical behaviour in various conditions. Additionally, the authors wish to acknowledge Andrés Lantada Díaz for his insightful remarks and knowledge sharing on smart polymers, and Shunichi Hayashi, SMP Technologies Inc., Tokyo, Japan, for providing the PU-SMP material”.

The authors state that the scientific conclusions are unaffected. This correction was approved by the Academic Editor. The original publication has also been updated.
